# Supplementary material for: Green Synthesis of Titanium Dioxide Nanoparticles Using Ocimum sanctum Leaf Extract: In Vitro Characterization and Its Healing Efficacy in Diabetic Wounds
Source: Molecules. 2022 Nov 9;27(22):7712. doi: 10.3390/molecules27227712 (PMC9699599; doi:10.3390/molecules27227712)
Supplement: Supplementary file 1 [file molecules-27-07712-s001.zip › molecules-1967051-supplementary.pdf]

Supplementary Table S1. Qualitative phytochemical screening of *Ocimum sanctum* extract

S/No	Bioactive phytochemicals	Test /Reagents	Result
**1**	Alkaloids	Mayer's reagent	+
**2**	Flavonoids	Alkaline reagent test	+
**3**	Saponins	Frothing test	+
**4**	Tannins	a) Lead acetate test<br>b) Ferric chloride test	+
**5**	Terpenoids & Steroids	Salkowski test	+
**6**	Phenols	Ferric chloride test	+
**7**	Anthraquinones	Bontrager's test	-
**8**	Proteins	Biuret's test	+
**9**	Carbohydrate	Felhing's test	+
**10**	Fixed oil or fatty acid	Spot test	+

Note: +Present, and - Absent.1

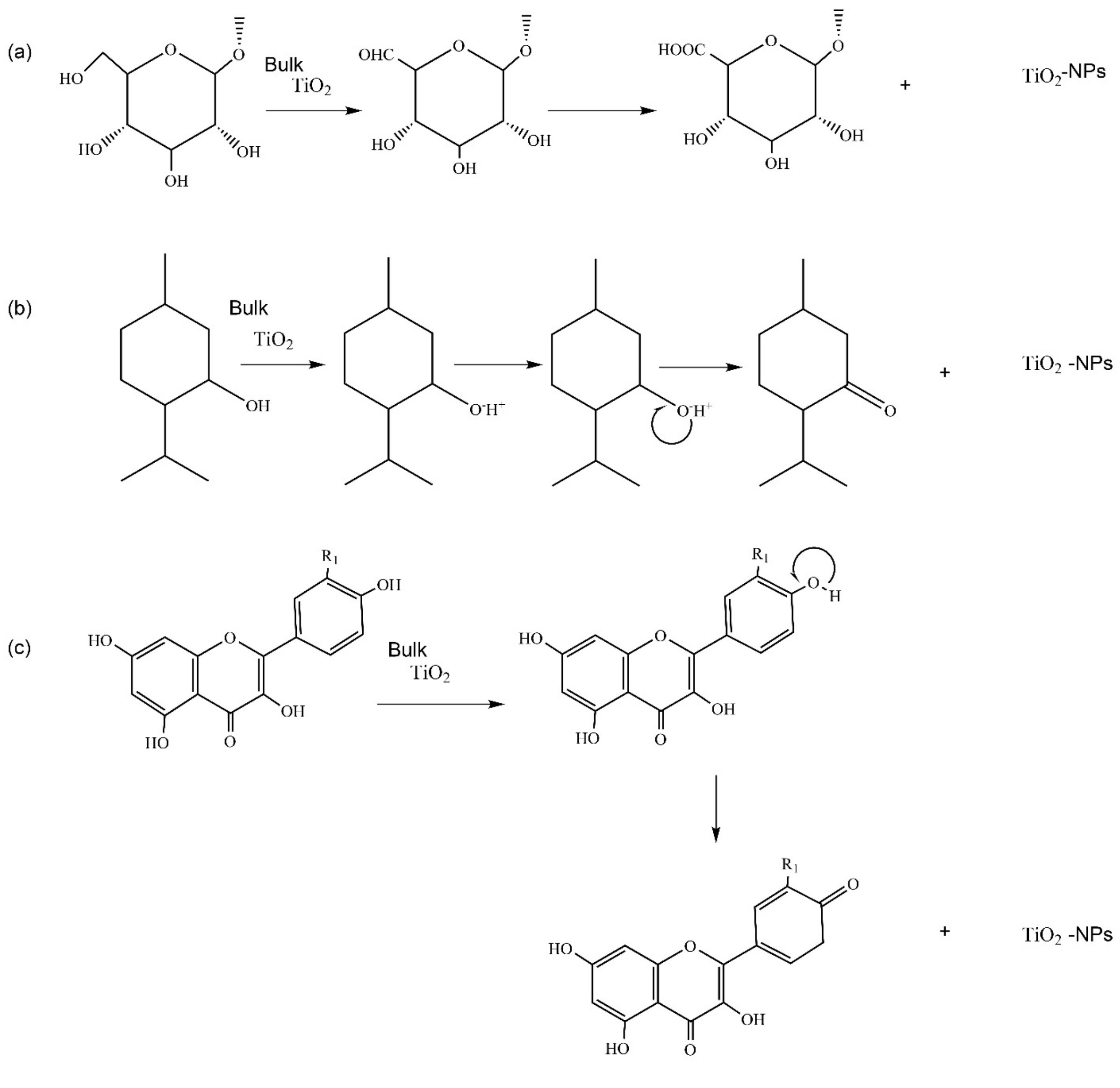

Supplementary Figure S1. Proposed bioproduction mechanism for TiO_2NPs by phytochemicals. (a). Biosynthesis of TiO_2 through saponins mediated bio-reduction. (b). Biosynthesis of TiO_2 through terpenoids mediated bio-reduction. (c). Biosynthesis of TiO_2 through flavonoids mediated bio-reduction.
